# Erythrocyte Membrane β-Adrenoreactivity as a Systemic Adrenergic Phenotype Associated with Ventricular Tachyarrhythmia in Heart Failure Patients Undergoing Cardiac Resynchronization Therapy

**DOI:** 10.3390/ijms27219912

**Published:** 2026-11-05

**Authors:** Tariel Atabekov, Elvira Muslimova, Tatyana Rebrova, Sergey Afanasiev, Roman Batalov, Sergey Popov

**Affiliations:** Cardiology Research Institute, Tomsk National Research Medical Center, Russian Academy of Sciences, Kievskaya St., 111a, Tomsk 634012, Russia; muslimovef@yandex.ru (E.M.); rebrova@cardio-tomsk.ru (T.R.); tursky@cardio-tomsk.ru (S.A.); romancer@cardio-tomsk.ru (R.B.); psv@cardio-tomsk.ru (S.P.)

**Keywords:** erythrocyte membrane β-adrenoreactivity, cardiac resynchronization therapy, ventricular tachyarrhythmia, heart failure with reduced ejection fraction, sympathetic nervous system, biomarker

## Abstract

Chronic hyperactivity of the sympathetic nervous system and remodeling of β-adrenergic receptors represent core pathophysiological mechanisms in heart failure with reduced ejection fraction (HFrEF) and in the emergence of ventricular arrhythmias. Nevertheless, clinically applicable functional indicators of this axis are still limited. We investigated whether erythrocyte membrane β-adrenoreactivity (EMA), an indirect functional membrane index derived from β-adrenergic antagonist-modulated hypo-osmotic hemolysis, correlates with a ventricular tachyarrhythmia (VTA) phenotype following cardiac resynchronization therapy with defibrillation capability (CRT-D). In a single-center observational cohort, 99 HFrEF patients underwent CRT-D implantation. A total of 70 patients with complete baseline and 12-month data formed the complete-case analysis (NCT03667989: n = 14; NCT05769036: n = 36; non-registry enrollment under the same institutional protocol: n = 20). Patients were categorized by the prespecified database variable “VTA preCRT and 12 m. post-CRT” into VTA-positive (n = 38) and VTA-negative (n = 32) groups. Baseline EMA was higher in VTA-positive patients (31.4% [22.8; 48.3]) than in VTA-negative patients (22.0% [15.8; 33.4]; *p* = 0.012). In multivariate logistic regression with stepwise selection, two variables were independently associated with the VTA-positive phenotype: EMA (odds ratio [OR] = 1.0446 per 1%; 95% confidence interval [CI] 1.0092–1.0812; *p* = 0.013) and left ventricular end-systolic dimension (LVESD) (OR = 1.1023 per 1 mm; 95% CI 1.0102–1.2029; *p* = 0.029). The model was statistically significant (likelihood-ratio χ^2^ = 12.567; *p* = 0.0019) and well calibrated (Hosmer–Lemeshow χ^2^ = 9.70; *p* = 0.286), with good discrimination (AUC = 0.748; 95% CI 0.629–0.844). However, explanatory power was modest (Nagelkerke R^2^ = 0.220), indicating that most variance in the VTA phenotype remains unexplained by EMA and LVESD. At a cut-off of 0.5, sensitivity was 73.68%, specificity 71.87%, and overall accuracy 72.86%. Internal validation with 2000 bootstrap iterations confirmed robustness (optimism-corrected AUC = 0.727; 95% CI 0.612–0.835). EMA remained significant after adjustment for LVESD, supporting its role as an independent marker of the VTA-positive phenotype. The model outperformed all univariate models in terms of AUC, R^2^, and overall accuracy. EMA may therefore be associated with a systemic adrenergic membrane phenotype and an arrhythmic substrate. These findings require mechanistic validation and external replication before any clinical application. All reported cut-offs are exploratory and derived from a single center, and the analysis is based on a complete-case cohort with acknowledged potential for attrition bias.

## 1. Introduction

Heart failure with reduced ejection fraction (HFrEF) is marked by sustained neurohormonal activation, adverse ventricular remodeling, electrical instability, and a persistent vulnerability to ventricular tachyarrhythmia (VTA) and sudden cardiac death, even with modern pharmacological and device-based therapies [1,2,3]. Heart failure affects an estimated 56–64 million people worldwide, with a global prevalence of approximately 1–2% in adults and rising to ≥10% among individuals older than 70 years. Population-attributable disability-adjusted life-years exceed 30 million annually, and roughly half of all HF patients have HFrEF [4,5]. These figures underscore the clinical and public-health relevance of identifying accessible biomarkers that refine arrhythmic risk stratification in this population. Cardiac resynchronization therapy (CRT) alleviates symptoms, enhances left ventricular (LV) performance, and improves clinical outcomes in appropriately chosen patients, yet the degree of benefit varies and is influenced by the electrical substrate, particularly QRS morphology and duration [2,6,7,8]. Notably, restoration of mechanical synchrony does not consistently eliminate arrhythmic risk [3,8,9,10].

The sympathetic nervous system connects progressive myocardial dysfunction to ventricular arrhythmogenesis. In the failing heart, persistent β-adrenergic stimulation triggers receptor desensitization, GRK2-mediated phosphorylation, β-arrestin recruitment, and receptor internalization and downregulation [7,10,11,12,13]. These events reprogram downstream signaling rather than merely terminating it, because β-arrestins also act as signaling scaffolds [12]. Compartment-specific cAMP signaling is maintained by phosphodiesterases, notably PDE4B and PDE4D, which differentially regulate cAMP microdomains near the plasma membrane, RyR2, and SERCA2a [14]. Disruption of this organization alters Ca^2+^ handling and facilitates diastolic Ca^2+^ leak. Prolonged catecholamine exposure also promotes PKA- and CaMKII-mediated RyR2 phosphorylation, increasing channel open probability and causing diastolic sarcoplasmic reticulum Ca^2+^ leak, which activates the electrogenic Na^+^/Ca^2+^ exchanger and promotes delayed afterdepolarizations [11,15,16]. Oxidative stress amplifies these processes by oxidizing RyR2 and activating CaMKII [16,17]. Collectively, these mechanisms reduce contractile reserve while creating a substrate for triggered activity and ventricular arrhythmia [7,10,11]. Autonomic indices measured by imaging, heart-rate variability, or implanted devices have also been associated with CRT response and VTA risk [1].

Erythrocyte membrane β-adrenoreactivity (EMA) is an experimentally accessible functional index based on the change in erythrocyte osmotic resistance produced by pharmacological blockade of membrane β-adrenergic receptors. In an earlier investigation involving individuals with coronary artery disease and implanted cardioverter–defibrillators, EMA was associated with later episodes of ventricular tachycardia [1]. Emerging evidence indicates that EMA is related to left ventricular systolic impairment and functional status in HF patients, whereby elevated values indicate diminished β-adrenoceptor sensitivity or density on the erythrocyte membrane [1,7,10]. Because erythrocytes lack cardiomyocyte T-tubules, sarcoplasmic reticulum, and excitation–contraction coupling, EMA cannot directly report PKA, CaMKII, RyR2, SERCA2a, or Na^+^/Ca^2+^ exchanger activity, or myocardial β1- or β2-adrenoceptor density. It should therefore be considered a peripheral functional membrane phenotype rather than a direct measure of myocardial β-adrenergic receptor biology. The inferential bridge is instead systemic: circulating catecholamines expose both erythrocytes and cardiomyocytes to the same neurohormonal milieu, and both cell types express β-adrenergic receptors coupled to Gs-cAMP signaling and subject to GRK2/β-arrestin-mediated desensitization. However, the evidence for desensitization in mature, anucleate erythrocytes is limited, and erythrocyte β-adrenergic receptor subtype predominance differs from that of cardiomyocytes. EMA is therefore best regarded as a peripheral functional readout of receptor availability or sensitivity, a systemic adrenergic membrane phenotype, rather than a direct myocardial measurement [1,10,18].

EMA is a peripheral membrane assay and cannot serve as a direct readout of cardiomyocyte excitation–contraction coupling: mature erythrocytes possess neither T-tubules nor sarcoplasmic reticulum, and they lack the intracellular signaling architecture (PKA, CaMKII, RyR2, SERCA2a, NCX) that couples β-adrenergic stimulation to Ca^2+^ handling in the heart. Accordingly, EMA is interpreted here as a systemic adrenergic membrane phenotype rather than as a myocardial measurement. The inferential bridge is instead systemic: circulating catecholamines expose both erythrocytes and cardiomyocytes to the same neurohormonal milieu, and both cell types express β-adrenergic receptors coupled to Gs–cAMP signaling and subject to GRK2/β-arrestin-mediated desensitization. Erythrocyte β-adrenoreactivity therefore serves as a peripheral functional readout of receptor availability/sensitivity, a systemic adrenergic membrane phenotype, rather than a direct myocardial measurement.

We therefore tested the hypothesis that baseline EMA is associated with the presence of a composite pre-/post-CRT VTA phenotype over 12 months after cardiac resynchronization therapy with defibrillation capability (CRT-D) implantation. We additionally evaluated whether the association with VTA persisted after adjustment for major clinical and electrical covariates and performed internal validation of the resulting association model.

## 2. Results

### 2.1. Study Cohort and Definition of the VTA Phenotype

**Figure 1:**
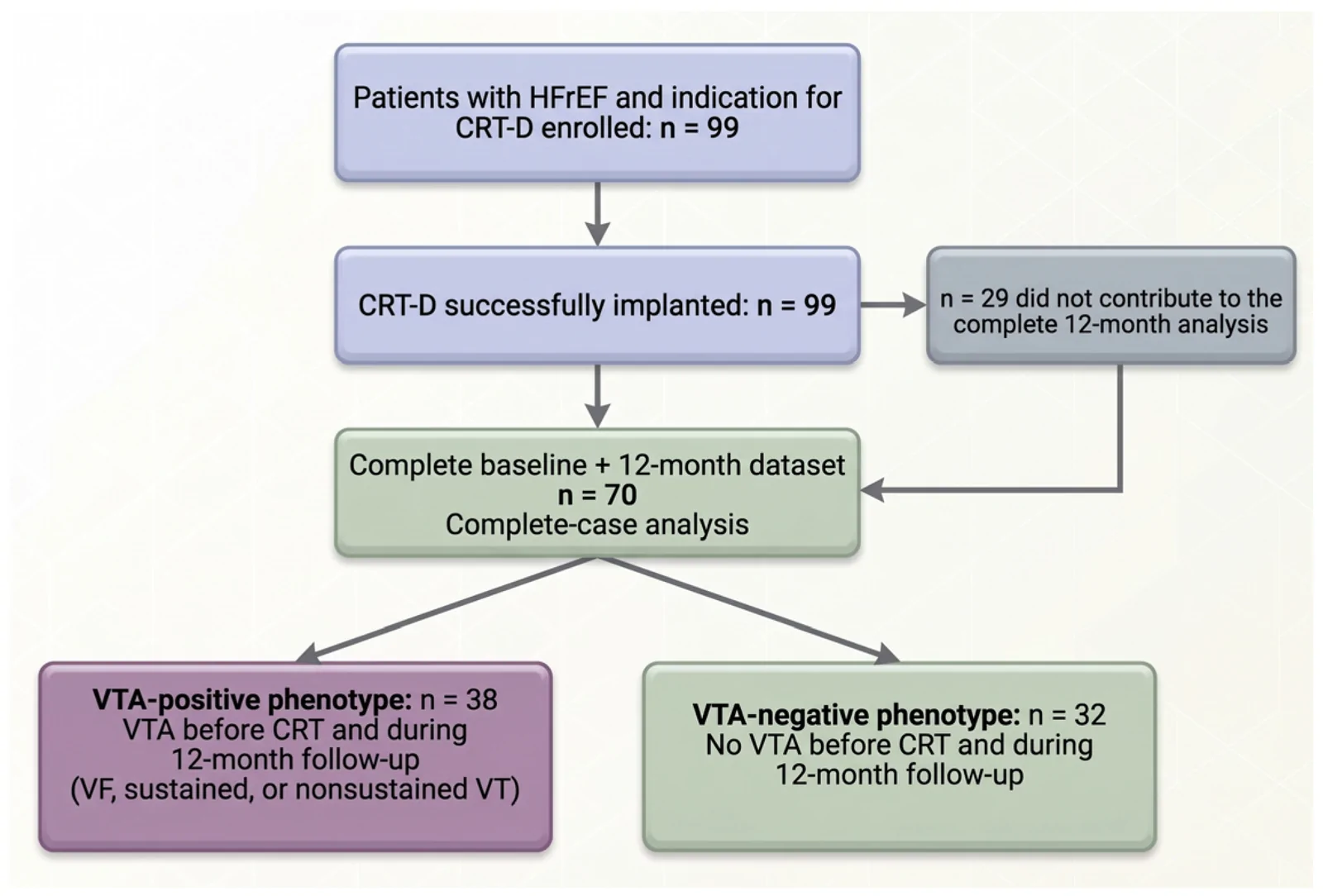
Study flow and definition of the ventricular tachyarrhythmia phenotype. HFrEF, heart failure with reduced ejection fraction; CRT-D, cardiac resynchronization therapy with defibrillation capability; VTA, ventricular tachyarrhythmia; VF, ventricular fibrillation; VT, ventricular tachycardia. Of 99 implanted patients, 29 did not contribute to the complete-case analysis. Reasons for exclusion were: death from a non-cardiovascular cause (n = 2), loss to follow-up/relocation (n = 7), incomplete 12-month echocardiographic dataset (n = 4), missing baseline blood samples for the EMA assay (n = 15), and withdrawal of consent (n = 1). Baseline data for the excluded patients were incomplete and were not systematically abstracted for the variables required for between-group comparison. The composition of the excluded group and the arrhythmia status of these patients, where ascertainable, are described in Section 2.1.

Ninety-nine patients with HFrEF and an indication for CRT-D underwent successful device implantation. Seventy patients had the complete baseline and 12-month dataset required for the present complete-case analysis. 29 patients did not contribute to the final analysis (Figure 1). The 29 patients were excluded from the complete-case analysis. The reasons for exclusion and the arrhythmia status of the excluded patients, where ascertainable from the available records, were as follows. Two patients died from non-cardiovascular causes. Neither had documented VTA before CRT-D implantation, and device-recorded electrograms could not be reviewed because the patients did not return for follow-up. Seven patients were lost to follow-up or relocated. Four of them had documented VTA before implantation and three had not, and post-implant electrograms were unavailable because these patients did not attend device checks. Four patients lacked complete 12-month echocardiographic data. Two had documented VTA both before and after implantation, and two had no VTA at either time point. These patients were excluded because a 12-month echocardiographic parameter was one of the prespecified predictors, and the baseline-to-12-month change in this parameter could not be ascertained. Fifteen patients lacked baseline blood samples for the EMA assay. Of these, seven had VTA before implantation, five had VTA after implantation, and three had VTA both before and after implantation. Six of these 15 patients also lacked the 12-month EMA sample. One patient withdrew consent 4 months after implantation. This patient had no history of VTA before CRT-D and did not attend follow-up, so post-implant electrograms were unavailable.

Because the baseline and follow-up records of the 29 excluded patients were incomplete and were not systematically abstracted for the variables required for a formal between-group comparison, we did not construct an analyzed-versus-excluded comparison table. We acknowledge that this precludes a formal assessment of the direction and magnitude of attrition bias, and we have therefore interpreted all findings as deriving from a complete-case cohort rather than from the full implanted population. This limitation is stated explicitly in Section 3.8.

Among the 70 analyzed patients, 38 were classified as VTA-positive and 32 as VTA-negative. The VTA-positive phenotype comprised patients with documented ventricular fibrillation, sustained ventricular tachycardia, or nonsustained ventricular tachycardia before CRT-D implantation and/or during the 12-month follow-up, according to the prespecified database grouping variable. Among the 38 VTA-positive patients, VTA was documented only before CRT-D implantation in 5, only after implantation in 18, and both before and after implantation in 15. Pre-implant VTA was therefore documented in 20 patients (5 with pre-implant-only VTA and 15 with VTA both before and after implantation). Among the 20 patients with pre-implant arrhythmias recorded in the database, the arrhythmia types were as follows: nonsustained VT in 8, nonsustained and sustained VT in 6, sustained VT in 4, sustained VT and VF in 1, and VF in 1 (Table 1). Consequently, the 54.3% VTA-positive proportion should be interpreted as the prevalence of a composite arrhythmic phenotype in the complete-case cohort, not as the 12-month incidence of new VTA.

The mean age of the analytical cohort was 60.3 ± 10.4 years, and 71.4% were male (Table 1). Baseline age, sex, blood pressure, body mass index, kidney function, heart failure etiology, major comorbidities, and most guideline-directed therapies were not significantly different between the two phenotype groups. New York Heart Association (NYHA) functional class III was more frequent in the VTA-positive group (55.2% vs. 25.0%; *p* = 0.015). Beta-blocker use was high and similar between groups (92.1% vs. 93.7%; *p* = 1.000), limiting the likelihood that the simple presence or absence of beta-blockade explains the EMA difference. Among treated patients, the distribution of beta-blocker agents and doses was comparable between the VTA-positive and VTA-negative groups: bisoprolol 2.5 mg, 31.6% vs. 28.1% (*p* = 0.798); bisoprolol 5 mg, 34.2% vs. 31.2% (*p* = 1.000); metoprolol 50 mg, 21.0% vs. 9.4% (*p* = 0.208); metoprolol 100 mg, 2.6% vs. 6.2% (*p* = 0.589); metoprolol 150 mg, 2.6% vs. 3.1% (*p* = 1.000); metoprolol 200 mg, 0.0% vs. 3.1% (*p* = 0.457); carvedilol 12.5 mg, 0.0% vs. 6.2% (*p* = 0.205); and carvedilol 25 mg, 0.0% vs. 6.2% (*p* = 0.205). Neither the distribution of agents nor the dose categories differed significantly between groups. However, beta-blocker type, dose, adherence, and treatment duration were not modeled as time-varying covariates, and residual confounding by treatment intensity cannot be excluded.

### 2.2. Baseline EMA, Functional Capacity, and Cardiac Structure

Table 2 summarizes baseline laboratory, electrocardiographic, and echocardiographic parameters. Notably, baseline EMA was significantly higher in the VTA-positive group than in the VTA-negative group (Table 2; *p* = 0.012), with an absolute median separation of 9.4 percentage points despite similar beta-blocker prevalence and no significant between-group differences in plasma potassium, glucose, total cholesterol, N-terminal pro-B-type natriuretic peptide (NT-proBNP), corrected QT interval, left ventricular ejection fraction (LVEF), or left ventricular end-systolic volume (LVESV). The 6-min walk test (6MWT) was lower in the VTA group (*p* = 0.033), consistent with greater functional impairment, and left ventricular end-systolic dimension (LVESD) and left ventricular sphericity index (LVSI) were larger in VTA-positive patients (*p* = 0.024 and *p* = 0.029, respectively), whereas interventricular septum (IVS) thickness was lower (*p* = 0.026).

### 2.3. Baseline-to-12-Month Changes After CRT-D

Both phenotype groups demonstrated substantial clinical and mechanical improvement after CRT-D (Table 3). NYHA class III was no longer present at 12 months in either group. European quality-of-life group questionnaire (EuroQoL EQ-5D) scores increased and Minnesota Living with Heart Failure Questionnaire (MLWHFQ) scores decreased markedly in both groups (all within-group *p* < 0.001). QRS duration shortened by approximately 37 ms in the VTA-positive group and 33 ms in the VTA-negative group (both *p* < 0.001). Six-minute walk distance increased from 280 to 450 m in the VTA-positive group and from 321 to 500 m in the VTA-negative group (both *p* < 0.001); the 12-month between-group difference remained significant (*p* = 0.006).

Mechanical reverse remodeling was evident in both groups. LVESD decreased from 60.2 ± 7.1 to 52.1 ± 11.6 mm in VTA-positive patients and from 56.7 ± 4.7 to 47.0 ± 10.2 mm in VTA-negative patients (both *p* < 0.001). LVESV decreased from 175 [145–210] to 116 [85–167] mL and from 159.5 [129.5–198] to 98 [67.5–121.5] mL, respectively (both *p* < 0.001). LVEF increased from 29 [25–32]% to 38 [33–44]% and from 30 [26–35]% to 42.5 [36–52.5]% (both *p* < 0.001). Global longitudinal strain also improved significantly in both groups. At 12 months, LVESV remained higher in VTA-positive patients (*p* = 0.045), while LVESD showed a borderline between-group difference (*p* = 0.059). Importantly, the between-group differences in change scores were not significant for LVESV (*p* = 0.255) or LVEF (*p* = 0.182); therefore, these data support reverse remodeling in both groups but do not establish a statistically different magnitude of remodeling between phenotypes.

EMA behaved differently from the structural and functional measures. In the VTA-positive group, EMA changed from 31.4 [22.8–48.3]% to 29.2 [18.8–42.7]% (*p* = 0.267); in the VTA-negative group, it changed from 22.0 [15.8–33.4]% to 24.1 [17.9–32.2]% (*p* = 0.667). The 12-month between-group comparison was no longer significant (*p* = 0.260), and the median change in EMA did not differ between groups (−1.4 [−15.6 to 5.9] vs. −0.1 [−5.6 to 8.6] percentage points; *p* = 0.255). Thus, the present data do not demonstrate that CRT-D systematically normalizes or shifts EMA. Rather, the principal signal is the baseline association with the VTA phenotype.

### 2.4. Exploratory Correlates of EMA

Across all 70 patients, EMA showed passive correlations with body mass index (Spearman R = 0.261; *p* = 0.029), MLWHFQ score (R = 0.319; *p* = 0.007), and NT-proBNP (R = 0.352; *p* = 0.026). Within the VTA-positive group, EMA correlated with MLWHFQ (R = 0.344; *p* = 0.034), QRS duration (R = −0.355; *p* = 0.028), NT-proBNP (R = 0.420; *p* = 0.046), and LV global longitudinal strain (R = −0.386; *p* = 0.046). In the VTA-negative group, EMA correlated with LV sphericity index (R = 0.460; *p* = 0.008) and LV global longitudinal strain (R = 0.467; *p* = 0.028).

These correlations are exploratory and should not be interpreted as evidence of a causal pathway. Several tests were performed in relatively small subgroups without a reported multiplicity adjustment, and some directions depend on measurement convention (notably the negative numerical scale used for global longitudinal strain). Their main value is hypothesis generation: EMA appears to covary with indices of symptom burden, neurohormonal stress, electrical activation, and ventricular geometry, but the pattern is not uniform across phenotypes.

### 2.5. Univariable Associations with the VTA Phenotype

In univariable logistic regression, baseline EMA was associated with the VTA-positive phenotype (odds ratio [OR] 1.0426 per 1 percentage-point increase; 95% confidence interval [CI] 1.0088–1.0774; *p* = 0.013). The corresponding area under the receiver-operating-characteristic curve (AUC) was 0.675 (95% CI 0.552–0.782), indicating only modest discrimination when EMA was used alone. At a probability threshold of 0.5, sensitivity was 65.8%, specificity 59.4%, and overall accuracy 62.9%. The Hosmer–Lemeshow statistic did not indicate gross lack of fit (χ^2^ = 5.75; *p* = 0.675), although this test has limited power in small samples.

Other individually significant variables included NYHA class III (OR 3.71; 95% CI 1.33–10.32; *p* = 0.012), LVESD (OR approximately 1.10 per mm; *p* = 0.030), LV sphericity index (OR 1.50 per 0.05-unit increase; *p* = 0.034), interventricular septal thickness (inverse association; OR 0.679 per mm; *p* = 0.032), and 6MWT (OR 0.93 per 10 m; *p* = 0.037). The 6MWT model showed evidence of poor calibration by the Hosmer–Lemeshow test (*p* = 0.048). These findings were therefore treated as candidate associations rather than stand-alone clinical classifiers. NT-proBNP, age, male sex, ischemic HF etiology, and amiodarone use were not statistically significant in univariate analysis.

### 2.6. Multivariable Model, Discrimination, Calibration, and Internal Validation

Stepwise multivariable logistic regression retained two variables: EMA and LVESD. EMA remained independently associated with the VTA-positive phenotype (adjusted OR 1.0446 per 1 percentage point; 95% CI 1.0092–1.0812; *p* = 0.013), as did LVESD (adjusted OR 1.1023 per 1 mm; 95% CI 1.0102–1.2029; *p* = 0.029). Expressed at clinically more intuitive scales, a 5-percentage-point higher EMA corresponds to approximately 24% higher odds of belonging to the VTA-positive phenotype (OR = 1.24), and a 10-point increase to approximately 55% higher odds (OR = 1.55), assuming log-linearity. Likewise, a 5-mm higher LVESD corresponds to approximately 63% higher odds (OR = 1.63), and a 10-mm increase to approximately 2.65-fold higher odds. These transformations describe the fitted association and should not be interpreted as causal effects or prospective event-risk increments.

The two-variable model was statistically significant overall (likelihood-ratio χ^2^ = 12.567; *p* = 0.0019) with Nagelkerke R^2^ = 0.220. Apparent discrimination was AUC = 0.748 (95% CI 0.629–0.844). At the conventional probability threshold of 0.5, sensitivity was 73.68%, specificity 71.87%, and overall accuracy 72.86%. The Youden-derived threshold (>0.49) produced sensitivity 76.32% and specificity 71.87%, with LR+ = 2.71 and LR− = 0.33. Because this threshold was derived in the same dataset, it should be considered exploratory rather than a clinically transportable cut-off.

ROC comparison showed that the combined model had a numerically higher AUC than EMA alone (0.748 vs. 0.675), but the AUC difference of 0.0728 was not statistically significant (DeLong *p* = 0.134) (Table 4). The combined model did outperform LVESD alone (AUC 0.639) in the pairwise comparison (difference 0.109; *p* = 0.045). Thus, the data support complementary information from the adrenergic membrane and structural domains, but they do not establish statistically significant incremental discrimination over EMA alone in this sample.

Calibration diagnostics were acceptable: the Hosmer–Lemeshow test was χ^2^ = 9.70 (*p* = 0.286), and the apparent Brier score was 0.192. The supplied bootstrap analysis used 2000 resamples to address optimism from model development and stepwise selection. EMA was retained in 84.5% and LVESD in 78.2% of bootstrap models. The apparent AUC of 0.748 was reduced by estimated optimism of 0.021 to an optimism-corrected AUC of 0.727 (bootstrap 95% bias-corrected and accelerated [BCa] CI 0.612–0.835). Optimism-corrected Nagelkerke R^2^ was 0.196 and the corrected Brier score was 0.208. Bootstrap BCa intervals remained above unity for both EMA (adjusted OR 1.044; 95% BCa CI 1.007–1.083) and LVESD (adjusted OR 1.101; 95% BCa CI 1.004–1.218).

Model-assumption checks supported internal numerical stability. The Box–Tidwell procedure found no significant departure from linearity in the logit for EMA or LVESD, and no complete or quasi-complete separation was identified. Cook’s distance values were <1, sensitivity analyses excluding minor influential observations did not materially change conclusions, and the EMA–LVESD linear association was negligible (R^2^ = 0.0013), yielding a variance inflation factor (VIF) of 1.0013 for both independent correlates. With 38 VTA-positive cases and two variables in the final model, the events-per-variable ratio was 19.0. These checks reduce, but do not eliminate, the risk of overfitting inherent in a small, single-center derivation sample.

To address potential instability from stepwise selection, we performed a sensitivity analysis using least absolute shrinkage and selection operator (LASSO) logistic regression with 10-fold cross-validation. The penalty parameter was selected by minimum binomial deviance. LASSO retained EMA and LVESD as the only variables with non-zero coefficients (penalized ORs: EMA 1.041 per 1%, LVESD 1.096 per 1 mm), consistent with the stepwise model. The LASSO-derived model achieved an AUC of 0.744 (95% CI 0.625–0.841), virtually identical to the stepwise model. These findings suggest that the two-variable solution is not an artifact of stepwise selection, although the small sample size still limits precision.

### 2.7. Assumption Verification and Multicollinearity Assessment

Formal assessment of multicollinearity revealed a negligible linear relationship between EMA and LVESD (R^2^ = 0.0013), resulting in a VIF of 1.0013 for both variables, far below the acceptable threshold of 5.0. Box–Tidwell tests confirmed linearity of the logit for continuous markers of the VTA-positive phenotype (*p* > 0.05 for non-linear terms). Diagnostic checks for influential cases (Cook’s distance < 1.0) and data separation confirmed model stability. No significant outliers were identified that altered study conclusions.

### 2.8. Exploratory Time-to-VTA Analysis (Descriptive)

For descriptive purposes, patients with VTA documented only before implantation (n = 5) were assigned an arbitrary event time of 1 month after implantation, because no post-implant event time exists for them. This assignment is arbitrary and means that the Kaplan–Meier curves should not be interpreted as estimates of post-implant arrhythmia-free survival. In a sensitivity analysis excluding these 5 patients, the between-group differences remained significant for the combined cut-off (log-rank *p* < 0.05) and for EMA alone (log-rank *p* < 0.05).

**Figure 2:**
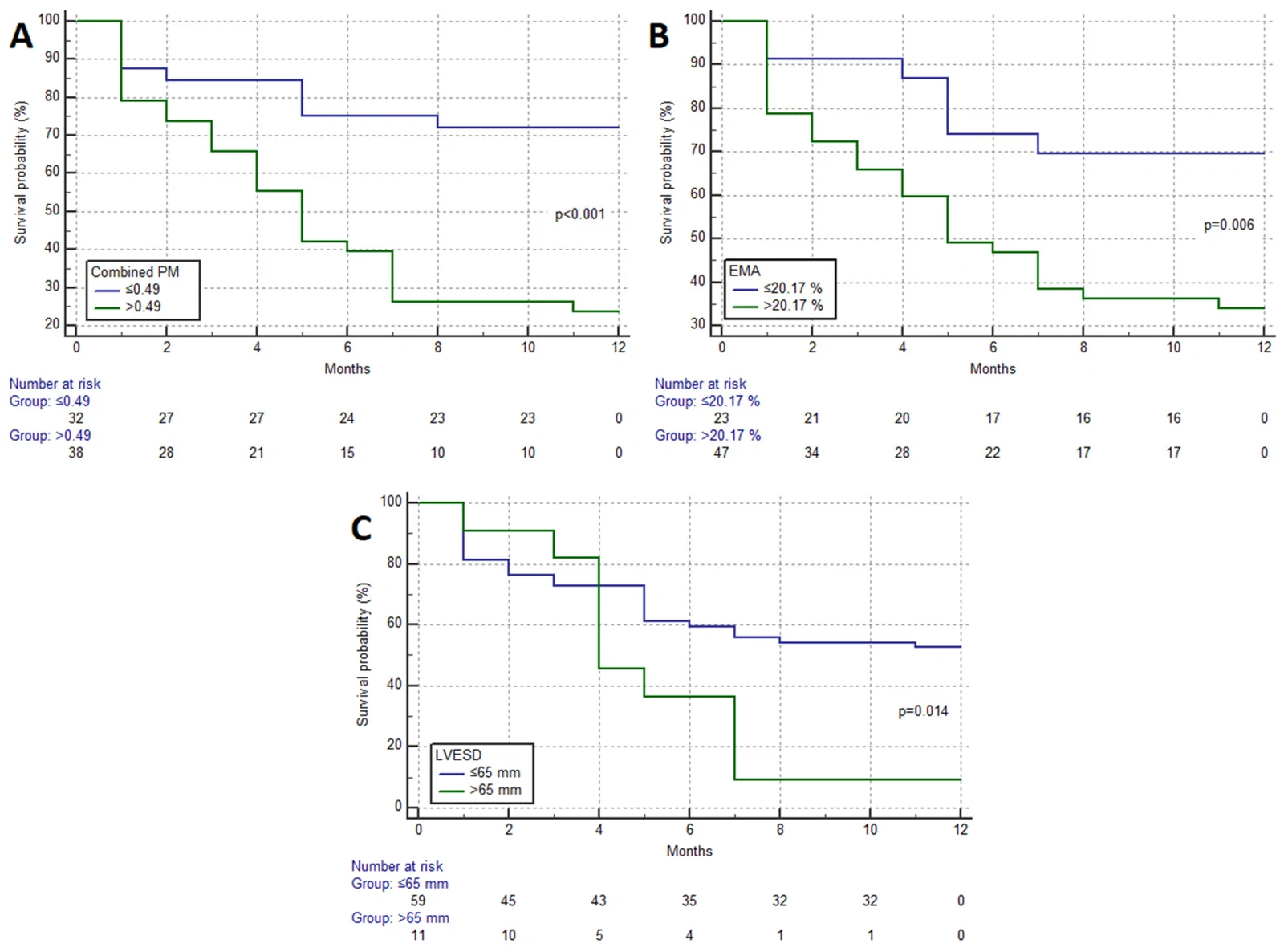
Exploratory Kaplan–Meier displays of time to VTA according to the combined EMA and LVESD cut-off (**A**), EMA alone (**B**), and LVESD alone (**C**). Patients with VTA documented only before implantation (n = 5) were assigned an arbitrary event time of 1 month. These curves are descriptive and do not represent post-implant arrhythmia-free survival. All cut-offs are exploratory and derived in the same dataset.

In the full cohort, the combined EMA and LVESD cut-off (>0.49) was associated with shorter time to VTA (n = 38; log-rank χ^2^ = 14.78; *p* = 0.0001; hazard ratio [HR] 3.604, 95% CI 1.925–7.516), as were the EMA cut-off alone (>20.17%; n = 47; χ^2^ = 7.40; *p* = 0.006; HR 2.789, 95% CI 1.309–5.241) and LVESD > 65 mm (n = 11; χ^2^ = 6.02; *p* = 0.014; HR 2.28, 95% CI 1.283–9.312) (Figure 2). After excluding the 5 patients with pre-implant-only VTA, the associations persisted (combined cut-off: n = 35, χ^2^ = 15.88, *p* = 0.0001, HR 4.354, 95% CI 2.111–8.957; EMA: n = 44, χ^2^ = 8.96, *p* = 0.003, HR 3.641, 95% CI 1.472–6.383; LVESD: n = 10, χ^2^ = 6.39, *p* = 0.011, HR 2.48, 95% CI 1.360–11.391). Hazard ratios were estimated by the Mantel-Haenszel method based on the log-rank statistic. These analyses are exploratory and descriptive, and the hazard ratios should not be interpreted as prospective risk estimates.

### 2.9. Exploratory Cox Proportional-Hazards Analysis in Patients Without Pre-Implant VTA

To avoid assigning arbitrary event times to patients with pre-implant-only VTA, we restricted this analysis to patients without any pre-implant VTA: the 18 patients with post-implant-only VTA and the 32 VTA-negative patients (n = 50). In this cohort, 18 patients had a first post-implant VTA event and 32 were censored. In an exploratory Cox proportional-hazards model including EMA and LVESD as the only covariates, EMA was associated with time to first post-implant VTA (HR 1.028 per 1%; 95% CI 1.003–1.054; *p* = 0.028), whereas LVESD was not (HR 1.029 per 1 mm; 95% CI 0.950–1.114; *p* = 0.485). The overall model did not reach the conventional significance threshold (likelihood-ratio χ^2^ = 5.390; df = 2; *p* = 0.068). Given the small number of events (18) and the exploratory nature of the analysis, these estimates should be interpreted cautiously. They do not establish a prospective time-to-event association and require validation in a larger, adequately powered cohort.

## 3. Discussion

### 3.1. Principal Findings

This study identifies two independent domains associated with the VTA-positive phenotype in HFrEF patients treated with CRT-D: a peripheral β-adrenergic membrane phenotype, represented by EMA, and structural ventricular remodeling, represented by LVESD. Baseline EMA was higher in VTA-positive patients, and the association persisted after adjustment for LVESD. The final two-variable model achieved moderate apparent discrimination (AUC 0.748) that remained similar after bootstrap correction (AUC 0.727), with stable variable-selection frequencies and minimal collinearity. At the same time, several observations constrain interpretation: VTA status combines pre-CRT and follow-up arrhythmias, the cohort is small and single-center, the model is internally rather than externally validated, and the combined model did not significantly improve AUC over EMA alone.

The longitudinal data provide a second important message. Both groups experienced robust electrical, functional, and mechanical improvement after CRT-D, yet baseline EMA itself did not change significantly within either group, and the between-group EMA difference was no longer significant at 12 months. Therefore, EMA should not be presented as a demonstrated marker of CRT response. In the present dataset, it behaves more plausibly as a baseline systemic membrane phenotype associated with an arrhythmic substrate than as a dynamic surrogate of reverse remodeling.

### 3.2. Molecular Interpretation: Chronic Sympathetic Drive and β-Adrenergic Receptor Remodeling

In HFrEF, sympathetic activation initially compensates for reduced stroke volume, but chronic catecholamine exposure becomes maladaptive [19,20,21]. β1-AR signaling in ventricular cardiomyocytes is predominantly coupled to Gs-adenylyl cyclase-cAMP signaling. Acute cAMP elevation activates protein kinase A (PKA) and exchange protein directly activated by cAMP, increasing L-type Ca^2+^ channel activity, RyR2-mediated sarcoplasmic reticulum Ca^2+^ release, phospholipid membrane phosphorylation, sarcoplasmic/endoplasmic reticulum calcium ATPase 2a (SERCA2a) function, and myofilament kinetics [22,23]. These effects increase contractile reserve but also increase energetic demand and the probability of spontaneous Ca^2+^ release when signaling is excessive or spatially dysregulated.

Chronic stimulation changes the receptor system itself through a coordinated molecular program. G protein-coupled receptor kinase 2 (GRK2) phosphorylates agonist-occupied β-adrenergic receptors, promotes β-arrestin recruitment, uncouples receptors from G proteins, and facilitates receptor internalization and downregulation [12,13]. Importantly, β-arrestins also serve as signaling scaffolds that activate mitogen-activated protein kinase cascades and other pathways, so receptor desensitization is not simply an “off” state but a reprogramming of downstream signaling [12]. Increased cardiac GRK2 in heart failure has been linked to impaired contractile signaling, apoptosis, fibrosis, metabolic dysfunction, and mitochondrial abnormalities [13]. The result is a paradoxical phenotype in which whole-body sympathetic drive is high while receptor responsiveness in selected compartments can be blunted or redistributed.

cAMP is also compartmentalized rather than uniformly distributed, and this spatial organization is enzymatically controlled. Phosphodiesterase 4B and phosphodiesterase 4D can differentially control cAMP near the plasma membrane, RyR2, and SERCA2a microdomains [14]. This spatial organization is biologically important because identical global cAMP concentrations can produce different local phosphorylation states and different arrhythmic consequences. Thus, a peripheral membrane assay such as EMA could theoretically capture one component of systemic receptor adaptation while remaining insensitive to the decisive subcellular cAMP architecture of cardiomyocytes. This is why EMA should be described as a functional peripheral membrane phenotype, not as a direct molecular measurement of myocardial β-adrenergic receptor density or signaling flux.

### 3.3. Adrenergic Signaling and Arrhythmic Susceptibility

Chronic β-adrenergic stimulation in failing myocardium is accompanied by Ca^2+^/calmodulin-dependent protein kinase II (CaMKII) activation, increased RyR2 diastolic open probability, sarcoplasmic reticulum Ca^2+^ leak, and Na^+^/Ca^2+^ exchange–mediated delayed afterdepolarizations, which can trigger ventricular tachycardia (VT) or fibrillation (VF) in a susceptible substrate [2,14,15,16,17,24]. These pathways are cited here only as established background: none of them was measured in the present study, and no direct mechanistic link between erythrocyte β-adrenoceptor reactivity and cardiomyocyte Ca^2+^ handling has been demonstrated. Because mature erythrocytes lack T-tubules, sarcoplasmic reticulum, excitation–contraction coupling, and innervation, EMA cannot directly report myocardial CaMKII, RyR2, or cAMP compartmentation. Its rationale rests instead on shared systemic catecholamine exposure and analogous receptor desensitization. Testing whether EMA tracks myocardial adrenergic remodeling would require paired erythrocyte and myocardial measurements of ADRB1/ADRB2 expression, GRK2, cAMP/PKA or CaMKII activity, RyR2 phosphorylation/oxidation, and catecholamine levels.

This mechanistic framework is presented as biological context, not as a demonstrated pathway in the present study. Neither myocardial CaMKII activity, RyR2 phosphorylation/oxidation, cAMP compartmentation, nor catecholamine levels were measured in our cohort; the proposed links between EMA and triggered activity are therefore inferential.

### 3.4. What Does EMA Measure?

EMA must be interpreted with care. Erythrocytes are anucleate and lack sympathetic innervation, and their β-adrenergic responsiveness reflects circulating catecholamines, receptor density and conformation, GRK2/β-arrestin-mediated desensitization, membrane lipid organization, and cumulative prior exposure rather than instantaneous sympathetic nerve activity. EMA is therefore a systemic adrenergic membrane phenotype, a cumulative peripheral readout, not a surrogate for cardiac sympathetic drive. Plasma catecholamines were not measured; although their future measurement would help determine whether EMA tracks systemic catecholamine exposure, plasma levels reflect multi-organ spillover and do not specifically index cardiac sympathetic activity. Cardiac sympathetic function is better assessed by ^123^I-MIBG imaging or device-derived indices, neither of which was available here.

Methodologically, EMA is derived from β-adrenergic antagonist modulation of hypo-osmotic erythrocyte hemolysis: a β-blocking compound is added to paired experimental samples before spectrophotometric measurement of hemoglobin release at 540 nm, and EMA is expressed as the ratio of optical-density signals from antagonist-exposed and control samples. Higher values are interpreted as lower membrane β-adrenergic receptor availability and/or sensitivity. Because this is a pharmacodynamic membrane assay, its signal may integrate receptor number, conformation, membrane lipid organization, downstream membrane-cytoskeletal responses, and other determinants of osmotic fragility.

Two caveats are essential. First, mature erythrocytes lack cardiomyocyte T-tubules, sarcoplasmic reticulum, excitation–contraction coupling, and cAMP microdomains; EMA therefore cannot directly report PKA, CaMKII, RyR2, SERCA2a, or Na^+^/Ca^2+^ exchanger activity. Second, the assay is not subtype-specific, so any inference to cardiac ADRB1 or ADRB2 biology is indirect; our group has previously shown that peripheral and myocardial measurements cannot be used interchangeably [18]. Notably, our findings run in the opposite direction to an earlier study in coronary disease with implanted cardioverter–defibrillators, in which lower EMA values correlated with VT/VF episodes [25]. This discrepancy argues against a universal monotonic threshold and likely reflects differences in substrate, medication, receptor adaptation, endpoint definition, timing, and the composite pre-/post-CRT phenotype used here. EMA should therefore be treated as a context-dependent functional biomarker requiring assay harmonization and external validation rather than applying the earlier 51.26% threshold to the present cohort.

The receptor biology of erythrocytes differs importantly from that of cardiomyocytes. Ventricular cardiomyocytes express predominantly β1-adrenergic receptors, with a smaller β2 population, whereas human erythrocytes express predominantly β2-adrenergic receptors, and their β-adrenergic signaling is coupled to Gs and cAMP accumulation [26,27]. This subtype difference means that EMA cannot be assumed to report myocardial β1-adrenergic receptor biology even in principle. In addition, mature erythrocytes are anucleate and transcriptionally silent, so classical agonist-induced receptor downregulation through transcriptional mechanisms cannot operate. Any change in erythrocyte β-adrenergic responsiveness must reflect post-translational processes, receptor conformational changes, membrane lipid and cytoskeletal organization, or cumulative exposure to circulating catecholamines rather than GRK2/β-arrestin-dependent transcriptional remodeling as described in cardiomyocytes. Direct evidence for GRK2/β-arrestin-mediated desensitization in mature human erythrocytes is limited, and we therefore do not claim that such a mechanism operates in erythrocytes. The analogy with cardiomyocyte desensitization is offered only as a plausible systemic parallel, not as a demonstrated shared pathway.

### 3.5. Structural Remodeling, LVESD, and the Arrhythmic Substrate

LVESD remained independently associated with the VTA phenotype after adjustment for EMA. This is biologically coherent because LV dilation is an integrated marker of chronic wall stress, myocyte loss or elongation, extracellular matrix remodeling, and altered ventricular geometry. Reverse remodeling is not merely a change in chamber size: it reflects coordinated changes in cardiomyocyte structure, fibrosis, metabolism, calcium handling, and neurohormonal load [28]. CRT can promote these changes by correcting electrical and mechanical dyssynchrony [28,29,30,31].

The present longitudinal data show substantial reverse remodeling in both phenotypes. QRS narrowed, LVESD and LVESV decreased, LVEF and strain improved, and functional capacity increased. This is consistent with the literature linking electrical remodeling to mechanical reverse remodeling after CRT [30] and with broader molecular and metabolic effects of resynchronization [32]. Nonetheless, improvement in pump function does not guarantee elimination of arrhythmic substrate. Ventricular scars, fibrosis, channel remodeling, and repolarization heterogeneity can persist, and CRT pacing itself changes activation and repolarization patterns [33]. Clinical data confirm that ventricular arrhythmia remains prognostically relevant after CRT, including among responders [34].

Our observation that 12-month LVESV remained higher in VTA-positive patients could reflect residual structural disease. However, the change in LVESV did not differ significantly between groups. It would therefore be inappropriate to conclude that the VTA-positive group had a poorer CRT remodeling response solely from the 12-month cross-sectional difference. A stronger analysis would model follow-up LV volume with adjustment for baseline LV volume or use prespecified longitudinal mixed models in a larger cohort.

Beyond device-based and pharmacological approaches, emerging biomaterials with excellent electrical conductivity and biocompatibility are being investigated as candidates for preventing post-infarct cardiac arrhythmias. Conductive hydrogels and bioengineered patches can bridge scarred myocardium, restore electrical continuity, and attenuate conduction heterogeneity—mechanisms conceptually complementary to CRT. Although these technologies are at a preclinical stage and were not evaluated in the present study, they highlight the need to invest resources in substrate-modifying strategies that may eventually complement or partially replace device-based rhythm management [35].

### 3.6. Integrating a Peripheral Adrenergic Phenotype with a Structural Substrate

The two retained model variables can be viewed as complementary biological domains. LVESD captures a relatively slow structural component of arrhythmic susceptibility, whereas EMA may capture a systemic membrane-adaptive component of sympathoadrenergic biology. Their near-zero collinearity in this dataset (VIF 1.0013) supports statistical complementarity, although it does not prove mechanistic independence. The combined model’s AUC was numerically higher than either marker alone and significantly higher than LVESD alone, but not significantly higher than EMA alone. Thus, the most defensible interpretation is that EMA carries information not explained by ventricular size and that combining the markers is promising, rather than that the two-marker model has already demonstrated decisive incremental clinical utility.

The exploratory correlations are directionally compatible with a systemic stress phenotype in some domains: higher EMA tracked with worse MLWHFQ score and higher NT-proBNP in the overall cohort. Yet the subgroup correlation structure was heterogeneous and some coefficients were counterintuitive or sensitive to measurement scale. Because many correlations were tested without multiplicity correction, they should be used to generate molecular hypotheses rather than to construct a post hoc causal network. Future studies should couple EMA with circulating catecholamines, myocardial or leukocyte GRK2, β-adrenergic receptor protein abundance, cAMP/PKA or CaMKII signatures, RyR2 phosphorylation/oxidation, fibrosis imaging, and device-derived VTA burden. That multimodal design would directly test whether EMA is a proxy for sympathetic exposure, receptor desensitization, membrane remodeling, or another process.

### 3.7. Statistical and Translational Interpretation

Several elements of the statistical analysis strengthen the internal interpretation. The final model is parsimonious, logit linearity was checked, influential observations and separation were assessed, collinearity was negligible, and bootstrap resampling quantified optimism. The corrected AUC of 0.727 indicates that discrimination is likely moderate rather than exceptional. The wide AUC confidence interval reflects the sample size and should temper claims of precision. Similarly, the apparent classification accuracy near 73% is dependent on prevalence and threshold and should not be extrapolated to other populations.

The data-derived Youden cut-off is particularly vulnerable to optimism and should not be proposed as a treatment threshold. The primary regression outcome is also not incident 12-month VTA because the phenotype includes events documented before CRT. Accordingly, terms such as “predictor of future VTA” should be avoided unless a new analysis is restricted to patients without pre-implant VTA and models time to first post-implant event. The current result is best described as an independent association with a VTA-positive phenotype. This wording preserves the biological signal without exceeding the study design.

Finally, the large number of baseline comparisons and exploratory correlations raises the possibility of false-positive findings. The regression result for EMA and LVESD remained statistically significant in bootstrap BCa intervals, which is reassuring, but internal resampling cannot correct all forms of selection bias, attrition bias, or center-specific measurement behavior. Replication should therefore focus on the pre-specified two-marker hypothesis rather than repeating broad data-driven selection.

### 3.8. Limitations

This study has several limitations. First, it is a single-center observational analysis with a complete-case sample of 70 of 99 implanted patients. Twenty-nine patients (29.3%) did not contribute to the 12-month analytical dataset. Their reasons for exclusion and, where ascertainable, their arrhythmia status are now reported in Section 2.1 and Figure 1. Baseline data for these patients were incomplete and were not systematically abstracted for the variables required for a formal between-group comparison. Consequently, an analyzed-versus-excluded comparison could not be constructed, and the direction and magnitude of potential attrition bias cannot be reliably estimated. Because two deaths occurred and because missingness may be informative with respect to arrhythmic risk, selection bias cannot be excluded, and the findings should be interpreted as deriving from a complete-case cohort rather than from the full implanted population. Second, the VTA-positive grouping combines pre-CRT and follow-up arrhythmia, preventing a clean prospective interpretation of the logistic model. Third, the sample is small, and stepwise selection can introduce coefficient bias and unstable variable choice despite an acceptable final events-per-variable ratio and 2000-iteration bootstrap validation. A LASSO sensitivity analysis yielded a consistent two-variable solution, but penalized regression cannot substitute for external validation. Fourth, multiple baseline and correlation tests were performed without a reported multiplicity correction. Fifth, EMA is a peripheral erythrocyte membrane functional assay and is not a direct measurement of myocardial β-adrenergic receptor subtype density, GRK2, β-arrestin, cAMP compartmentation, PKA, CaMKII, RyR2 function, catecholamine concentration, or myocardial fibrosis. Sixth, beta-blocker type and dose were recorded and compared between groups, but treatment intensity was not modeled as a time-varying covariate. Seventh, the composite VTA phenotype combines nonsustained VT, sustained VT, and VF, which differ in clinical severity. Eighth, all model cut-offs and performance estimates are derived from the same center and require prospective external validation before clinical use.

## 4. Materials and Methods

### 4.1. Study Design and Population

The study followed a non-randomized, single-center observational cohort design. Its population comprised HFrEF patients who had undergone CRT-D implantation at the Department of Surgical Arrhythmology and Cardiac Pacing, Cardiology Research Institute, from October 2021 through July 2025. Of the 99 consecutive patients enrolled and successfully implanted, 70 met the criteria for the present complete-case analysis, which required a full baseline and 12-month dataset covering clinical, electrocardiographic, echocardiographic, device, and laboratory variables. Recruitment of these participants was conducted within registered clinical trials: ClinicalTrials.gov NCT03667989 (registered 11 September 2018) and NCT05769036 (registered 20 February 2023). Patients were enrolled consecutively under two registered protocols (NCT03667989, n = 14; NCT05769036, n = 36) and, for the remaining 20 patients, under the same institutional protocol outside the trial registries. The study was exploratory and hypothesis-generating. No a priori power calculation was performed. The analytical cohort was defined by the availability of a complete baseline and 12-month dataset, and the sample size was therefore determined by the number of consecutively implanted patients who completed 12-month follow-up with all required measurements. This is acknowledged as a limitation (Section 3.8), and the findings should be interpreted as deriving from a complete-case cohort rather than from the full implanted population.

The source protocol defined the eligible population as patients with HFrEF, LVEF ≤35%, sinus rhythm, and a prolonged QRS complex consistent with CRT indications (QRS ≥130 ms in those with left bundle branch block morphology and ≥150 ms in those without it). Individuals were not eligible if any of the following applied: age ≤18 years; NYHA class I or IV; hypertrophic cardiomyopathy; persistent atrial fibrillation; decompensated heart failure; myocardial infarction within the preceding 3 months; acute myocarditis; prior pacemaker or cardioverter–defibrillator implantation; pregnancy; cognitive impairment that would interfere with participation; a need for revascularization or heart transplantation; a major comorbidity that could prevent protocol completion; or missing complete 12-month clinical, echocardiographic, or medical-therapy data. Heart failure etiology was assigned to one of three categories (ischemic, non-ischemic, or mixed) on the basis of coronary anatomy, a history of myocardial infarction or revascularization, and other available etiologic investigations.

### 4.2. Clinical Follow-Up and VTA Phenotype

Baseline and follow-up evaluations consisted of a clinical assessment, 6-min walk testing, 12-lead electrocardiography (ECG), Holter monitoring, transthoracic echocardiography, speckle-tracking echocardiography, blood testing (including NT-proBNP where available), and EMA measurement. Ventricular arrhythmia events recorded by the CRT-D device were extracted from device interrogation during follow-up.

In this analysis, VTA encompassed ventricular fibrillation and ventricular tachycardia, both sustained and nonsustained. Nonsustained VT was specified as ≥3 consecutive ventricular beats at ≥120 beats/min lasting < 30 s and sustained VT was specified as lasting ≥30 s, in accordance with current ventricular-arrhythmia definitions [33]. Pre-implant VTA was identified from ECG or Holter recordings, while post-implant VTA was identified from ECG, Holter, and stored CRT-D electrograms. According to the primary grouping variable, patients were considered VTA-positive when VTA had been documented before implantation and/or during the 12-month follow-up and VTA-negative when no VTA had been documented in either period.

### 4.3. ECG, Echocardiography, and Speckle-Tracking Analysis

ECG data were collected using standard 12-lead recordings (Nihon Kohden Corporation, Shinjuku-ku, Tokyo, Japan) obtained from supine patients at a paper speed of 25 mm/s and a gain of 10 mm/mV. The analysis focused on QRS duration and on a QT interval corrected for bundle branch block or biventricular pacing, as specified by the institutional protocol. Holter ECG monitoring was employed to detect arrhythmias and to provide supplementary rhythm evaluation.

Echocardiographic assessment via the transthoracic approach covered chamber dimensions and volumes, LVEF, valvular function, and routine hemodynamic parameters. LVESD and LVESV served as the structural remodeling variables. Speckle-tracking echocardiography was performed on an Affinity 70 CV system (Philips Ultrasound, Bothell, WA, USA), and the data were post-processed with QLAB version 15.7. Apical four-, two-, and three-chamber views were obtained at approximately 60–110 frames/s whenever feasible, and LV global longitudinal strain was calculated following the laboratory protocol. All analyses described in this manuscript were based on baseline and 12-month measurements.

### 4.4. Erythrocyte Membrane β-Adrenoreactivity Assay

EMA was assessed with a hypo-osmotic hemolysis assay using the BETA-ARM AGAT reagent kit (Agat-Med, Moscow, Russia). Venous blood was drawn into K2-EDTA-containing tubes. Whole blood was first diluted in isotonic saline; aliquots were then distributed into paired experimental and control hypo-osmotic buffer samples. The experimental samples were supplemented with 0.1 mL of a β-adrenergic antagonist solution (1-(1-isopropylamino)-3-(1-naphthalenyloxy)-2-propanol hydrochloride, 7.5 g/L), while 0.1 mL of distilled water was added to the control samples. After 0.05 mL of the prepared blood sample had been introduced, the samples were mixed gently without foaming, incubated at room temperature in accordance with the kit protocol, and centrifuged for 10 min at 1500 rpm. Supernatant optical density was read at 540 nm on a Biochrom WPA Biowave II spectrophotometer (Biochrom Ltd., Cambridge, Cambridgeshire, UK).

The assay exploits antagonist-associated modification of erythrocyte osmotic resistance. EMA (%) was calculated from duplicate experimental (Ee1, Ee2) and control (Ec1, Ec2) optical-density measurements using the equation: EMA = [(Ee1 + Ee2)/(Ec1 + Ec2)] × 100. Within the assay framework, higher EMA values were interpreted as reduced β-adrenergic membrane responsiveness, indicating lower receptor availability and/or sensitivity. Because the assay is performed in erythrocytes, this interpretation is peripheral and functional; it does not identify myocardial β-AR subtype density or downstream cardiomyocyte signaling.

### 4.5. CRT-D Implantation and Programming

CRT-D devices were implanted via the transvenous route with fluoroscopic assistance. The right ventricular defibrillation lead and the atrial lead, both active-fixation types, were positioned at the discretion of the operator. Where anatomically suitable, the LV lead was advanced into a lateral, posterolateral, or anterolateral coronary venous branch. Intraoperative checks confirmed proper lead placement and electrical function. The device was programmed following institutional and contemporary recommendations [29,36], with the following zones: a monitoring zone at approximately 140–170 beats/min without therapy; a VT therapy zone at approximately 170–200 beats/min providing antitachycardia pacing and shock capability; and a VF zone above approximately 200 beats/min offering antitachycardia pacing during charging together with high-energy shock therapy.

### 4.6. Statistical Analysis

Continuous measurements were expressed as mean ± standard deviation for parametrically distributed data and as median [interquartile range] for nonparametrically distributed data. Frequencies and percentages, n (%), were used to describe categorical measurements. Between-group differences in continuous variables were tested with the independent-samples t-test or Mann–Whitney U test, as dictated by data distribution, whereas Fisher’s exact test was applied to categorical variables. Changes within groups from baseline to 12 months were evaluated using paired-samples t-tests or Wilcoxon signed-rank tests, as appropriate; for paired categorical data, McNemar’s test was employed. Exploratory associations were examined with Spearman correlation coefficients. A two-sided *p*-value below 0.05 was considered nominally significant. Given the absence of multiplicity correction for the exploratory baseline and correlation analyses, the results of these analyses were interpreted with caution.

Binary logistic regression was used to examine associations with the VTA-positive phenotype. Univariable models were evaluated first. A stepwise multivariable model was then developed from candidate clinical and instrumental variables, with the final model retaining EMA and LVESD. ORs with 95% confidence intervals were reported. For continuous markers of the VTA-positive phenotype, linearity in the logit was assessed using the Box–Tidwell procedure. Multicollinearity was assessed with VIF. Complete/quasi-complete separation and influential observations were evaluated using cross-tabulations, standard-error behavior, Cook’s distance, difference in betas, leverage, and sensitivity analyses.

Model performance was characterized by the likelihood-ratio test, Cox–Snell and Nagelkerke R^2^, discrimination by receiver-operating-characteristic (ROC) analysis, and calibration by the Hosmer–Lemeshow test and Brier score. ROC curves were compared using DeLong’s method. Classification metrics were calculated at *p* = 0.5 and, exploratorily, at the Youden-derived threshold. To quantify overfitting and optimism associated with model development, internal validation used 2000 bootstrap resamples. Optimism-corrected AUC, Nagelkerke R^2^, Brier score, classification metrics, variable-selection frequencies, and BCa 95% confidence intervals were obtained from the supplied bootstrap analysis. Kaplan–Meier survival analysis with log-rank test was used to compare VTA-free survival across risk groups. Hazard ratios with 95% confidence intervals were calculated using the Mantel-Haenszel method based on the log-rank statistic. Two at-risk populations were used in the time-to-event analyses. The Kaplan–Meier sensitivity analyses in Section 2.8 excluded only the 5 patients with pre-implant-only VTA (n = 65) and retained the 15 patients with VTA both before and after implantation, for whom the post-implant event time is known. The Cox analysis in Section 2.9 excluded all 20 patients with any pre-implant VTA (n = 50), because a valid time-to-first-post-implant-event model requires that no event have occurred before the time origin. Statistical significance was set at *p* < 0.05. Analyses were performed using Statistica 10.0 (StatSoft, Tulsa, OK, USA) and MedCalc 19.2.6 (MedCalc Software Ltd., Ostend, West Flanders, Belgium).

### 4.7. Ethics

The present investigation was carried out in compliance with the Declaration of Helsinki and Good Clinical Practice requirements. The study was approved by the local ethics committee at the Cardiology Research Institute (Protocol No. 219, 26 October 2021, and Protocol No. 240, 15 February 2023). Fourteen patients were enrolled under NCT03667989, 36 under NCT05769036, and 20 outside the trial registries under the same institutional protocol and ethics approval. Informed consent was obtained from all participants. Each subject gave consent for publication of clinical data, consistent with the source documentation.

## 5. Conclusions

In HFrEF patients undergoing CRT-D, higher baseline erythrocyte membrane β-adrenoreactivity and larger LV end-systolic dimension were independently associated with a composite VTA-positive phenotype. The result supports a biologically plausible two-domain framework in which a peripheral sympathoadrenergic membrane phenotype complements a structural ventricular remodeling marker. The molecular context linking chronic sympathetic drive to arrhythmogenesis involves β-adrenergic receptor desensitization, GRK2-mediated phosphorylation, β-arrestin recruitment, compartmentalized cAMP signaling, CaMKII activation, RyR2 phosphorylation and oxidation, diastolic Ca^2+^ leak, and oxidative stress, all of which converge on triggered activity and reentrant substrate. The association remained internally stable after bootstrap correction, but discrimination was moderate and external validation is required. Because the outcome includes pre-implant VTA and EMA is an erythrocyte-based functional assay rather than a direct myocardial molecular measurement, the findings should not be interpreted as proof that EMA predicts incident VTA or directly quantifies cardiac β-adrenergic receptor remodeling. Prospective studies integrating EMA with myocardial or circulating molecular markers of β-AR/GRK2/cAMP/CaMKII/RyR2 signaling and strictly incident post-CRT arrhythmia endpoints are needed. All performance metrics, discrimination values, and risk cut-offs were derived from a single center and require prospective external validation before clinical implementation.

In HFrEF patients undergoing CRT-D, baseline erythrocyte membrane β-adrenoreactivity was independently associated with a composite VTA-positive phenotype after adjustment for LVESD, and the two-variable model showed moderate, internally validated discrimination. These results are exploratory, derive from a single-center complete-case cohort of 70 patients, and are subject to potential attrition and selection bias. They do not establish EMA as a predictor of incident post-implant VTA, and external validation in prospectively followed cohorts with minimal attrition is required before any clinical application.

## Figures and Tables

**Table 1:** Patient’s baseline characteristics according to the VTA phenotype.

Characteristics	Total (*n* = 70)	VTA+ (*n* = 38)	VTA− (*n* = 32)	Test Statistics	*p*_2–3_
	1	2	3		
Age, year, M ± SD	60.3 ± 10.4	60.5 ± 9.7	60.1 ± 11.3	t = −0.182	0.855
Male gender, *n* (%)	50 (71.4)	25 (65.8)	25 (78.1)	FET	0.297
Arterial hypertension, *n* (%)	2 (2.9)	2 (5.2)	0 (0.0)	FET	0.496
Heart failure etiology:					
Ischemic, *n* (%)	19 (27.1)	9 (23.7)	10 (31.2)	FET	0.591
Non-ischemic, *n* (%)	37 (52.9)	23 (60.5)	14 (43.7)	FET	0.229
Ischemic and non-ischemic, *n* (%)	14 (20.0)	6 (15.8)	8 (25.0)	FET	0.381
Coronary artery interventions in anamnesis:					
Stenting, *n* (%)	14 (20.0)	6 (15.8)	8 (25.0)	FET	0.381
Stenting and bypass grafting, *n* (%)	6 (8.6)	3 (7.9)	3 (9.4)	FET	1.000
Family history of CAD, *n* (%)	6 (8.6)	4 (10.5)	2 (6.2)	FET	0.681
Family history of SCD, *n* (%)	1 (1.4)	0 (0.0)	1 (3.1)	FET	0.457
SBP, mmHg, M ± SD	120.0 ± 9.6	120.5 ± 9.6	119.4 ± 9.7	t = −0.440	0.661
DBP, mmHg, M ± SD	74.8 ± 8.6	75.7 ± 5.8	73.8 ± 7.4	t = −1.207	0.231
Body mass index, kg/m^2^, M ± SD	28.4 ± 5.5	28.5 ± 6.1	28.2 ± 4.7	t = −0.228	0.819
eGFR, ml/min/1.73 m^2^, M ± SD	74.3 ± 18.3	75.6 ± 17.5	72.7 ± 19.5	t = −0.638	0.525
New York Heart Association functional class:					
II, *n* (%)	41 (58.6)	17 (44.7)	24 (75.0)	FET	0.015
III, *n* (%)	29 (41.4)	21 (55.2)	8 (25.0)	FET	0.015
Pre-implantation cardiac arrhythmias and conduction disease:					
Ventricular tachyarrhythmias, *n* (%):	20 (28.6)	20 (52.6)	0 (0.0)	FET	<0.001
Nonsustained VT, *n* (%)	8 (11.4)	8 (21.0)	0 (0.0)	FET	0.006
Nonsustained and sustained VT, *n* (%)	6 (8.6)	6 (15.8)	0 (0.0)	FET	0.028
Sustained VT, *n* (%)	4 (5.7)	4 (10.5)	0 (0.0)	FET	0.120
Sustained VT and VF, *n* (%)	1 (1.4)	1 (2.6)	0 (0.0)	FET	1.000
VF, *n* (%)	1 (1.4)	1 (2.6)	0 (0.0)	FET	1.000
Paroxysmal atrial fibrillation, *n* (%)	19 (27.1)	8 (21.0)	11 (34.3)	FET	0.282
Paroxysmal SVT, *n* (%)	2 (2.9)	1 (2.6)	1 (3.1)	FET	1.000
AVB 2-3 degrees, *n* (%)	8 (11.4)	6 (15.8)	2 (6.2)	FET	0.275
LBBB, *n* (%)	65 (92.9)	36 (94.7)	29 (90.6)	FET	0.654
RBBB, *n* (%)	5 (7.1)	2 (5.2)	3 (9.4)	FET	0.654
ICD, *n* (%)	2 (2.9)	1 (2.6)	1 (3.1)	FET	1.000
Comorbidities:					
Dyslipidemia, *n* (%)	34 (48.6)	20 (52.6)	14 (43.7)	FET	0.482
Diabetes mellitus, *n* (%)	15 (21.4)	9 (23.7)	6 (18.7)	FET	0.772
Stroke, *n* (%)	3 (4.3)	1 (2.6)	2 (6.2)	FET	0.589
COPD, *n* (%)	6 (8.6)	4 (10.5)	2 (6.2)	FET	0.681
Smoke, *n* (%)	17 (24.3)	10 (26.3)	7 (21.9)	FET	0.782
Therapy:					
Statins, *n* (%)	62 (88.6)	33 (86.8)	29 (90.6)	FET	0.719
Beta-blockers, *n* (%):	65 (92.9)	35 (92.1)	30 (93.7)	FET	1.000
Metoprolol 50 mg, *n* (%)	11 (15.7)	8 (21.0)	3 (9.4)	FET	0.208
Metoprolol 100 mg, *n* (%)	3 (4.3)	1 (2.6)	2 (6.2)	FET	0.589
Metoprolol 150 mg, *n* (%)	2 (2.9)	1 (2.6)	1 (3.1)	FET	1.000
Metoprolol 200 mg, *n* (%)	1 (1.4)	0 (0.0)	1 (3.1)	FET	0.457
Bisoprolol 2.5 mg, *n* (%)	21 (30.0)	12 (31.6)	9 (28.1)	FET	0.798
Bisoprolol 5 mg, *n* (%)	23 (32.9)	13 (34.2)	10 (31.2)	FET	1.000
Carvedilol 12.5 mg, *n* (%)	2 (2.9)	0 (0.0)	2 (6.2)	FET	0.205
Carvedilol 25 mg, *n* (%)	2 (2.9)	0 (0.0)	2 (6.2)	FET	0.205
ACEIs, *n* (%)	18 (25.7)	12 (31.6)	6 (18.7)	FET	0.278
Anticoagulants, *n* (%)	23 (32.9)	11 (28.9)	12 (37.5)	FET	0.610
Antiplatelet agents, *n* (%)	36 (51.4)	23 (60.5)	13 (40.6)	FET	0.149
MRAs, *n* (%)	67 (95.7)	38 (100.0)	29 (90.6)	FET	0.090
SGLT2Is, *n* (%)	60 (85.7)	35 (92.1)	25 (78.1)	FET	0.168
Loop diuretics, *n* (%)	32 (45.7)	18 (47.3)	14 (43.7)	FET	0.813
ARNI, *n* (%)	49 (70.0)	28 (73.7)	21 (65.6)	FET	0.601
Ivabradine, *n* (%)	4 (5.7)	2 (5.2)	2 (6.2)	FET	1.000
Amiodarone, *n* (%)	31 (44.3)	20 (52.6)	11 (34.3)	FET	0.152
Calcium channel blockers, *n* (%)	3 (4.3)	2 (5.2)	1 (3.1)	FET	1.000

Continuous variables are presented as mean ± standard deviation (SD) or as median (Me) with interquartile range [IQR; Q1–Q3], based on their distribution. Categorical variables are reported as absolute frequencies (n) with relative frequencies (%). Abbreviations: ACEIs, angiotensin-converting enzyme inhibitors; ARNI, angiotensin receptor neprilysin inhibitor; AVB, atrioventricular block; CAD, coronary artery disease; COPD, chronic obstructive pulmonary disease; DBP, diastolic blood pressure; eGFR, estimated glomerular filtration rate; ICD, intraventricular conduction disturbance; FET, Fisher’s exact test; LBBB, left bundle branch block; MRAs, mineralocorticoid receptor antagonists; pts, patients; RBBB, right bundle branch block; SBP, systolic blood pressure; SCD, sudden cardiac death; SGLT2Is, sodium glucose co-transporter 2 inhibitors; SVT, supraventricular tachycardia; t, Student’s t-statistic; U, inversion number criterion; VF, ventricular fibrillation; VT, ventricular tachycardia; VTA, ventricular tachyarrhythmia. Between-group comparisons were performed using the t-test or Mann–Whitney U test for continuous variables, as appropriate, and Fisher’s exact test for categorical variables. Pre-implant arrhythmia categories are listed for the 20 patients with any pre-implant VTA (5 with pre-implant-only VTA and 15 with VTA both before and after implantation). Categories are mutually exclusive with respect to arrhythmia type but not with respect to the post-implant classification.

**Table 2:** Patient’s baseline laboratory, electrocardiographic and echocardiographic characteristics according to the VTA phenotype.

Characteristics	Total (*n* = 70)	VTA+ (*n* = 38)	VTA− (*n* = 32)	Test Statistics	*p*_2–3_
	1	2	3		
Plasma potassium, mmol/L, M ± SD	4.5 ± 0.4	4.5 ± 0.4	4.5 ± 0.4	t = −0.011	0.991
Blood glucose, mmol/L, Me [Q1; Q3]	5.5 [5.0; 5.9]	5.4 [5.0; 5.9]	5.7 [5.1; 6.0]	U = 517.5	0.288
TC, mmol/L, Me [Q1; Q3]	3.9 [3.4; 4.8]	4.1 [3.4; 4.9]	3.9 [3.4; 4.4]	U = 570.0	0.658
NTproBNP, pg/mL, Me [Q1; Q3]	645.2 [239.5; 2107.0]	872.0 [257.0; 2598.0]	419.6 [141.9; 1607.0]	U = 160.0	0.338
EMA, %, Me [Q1; Q3]	27.7 [17.1; 46.2]	31.4 [22.8; 48.3]	22.0 [15.8; 33.4]	U = 395.5	0.012
QRS duration, ms, M ± SD	176.9 ± 20.6	180.7 ± 19.8	172.4 ± 21.1	t = −1.677	0.098
cQTc, ms, M ± SD	428.7 ± 26.6	428.9 ± 26.7	428.4 ± 26.9	t = −0.066	0.947
6MWT, m, Me [Q1; Q3]	315.0 [260.0; 350.0]	280.0 [250.0; 350.0]	321.0 [299.5; 400.0]	U = 426.5	0.033
LVEF, %, Me [Q1; Q3]	30.0 [25.0; 34.0]	29.0 [25.0; 32.0]	30.0 [26.0; 35.0]	U = 506.5	0.234
LVESV, ml, Me [Q1; Q3]	169.0 [139.0; 200.0]	175.0 [145.0; 210.0]	159.5 [129.5; 198.0]	U = 501.0	0.209
LVESD, mm, M ± SD	58.6 ± 6.3	60.2 ± 7.1	56.7 ± 4.7	t = −2.310	0.024
LVSI, mm, M ± SD	0.67 ± 0.07	0.68 ± 0.07	0.65 ± 0.06	t = −2.225	0.029
IVS, mm, M ± SD	9.9 ± 1.5	9.6 ± 1.4	10.3 ± 1.4	t = 2.274	0.026
LVGLS, %, Me [Q1; Q3]	−7.9 [−11.0; −6.8]	−7.8 [−10.1; −6.7]	−9.0 [−11.2; −6.9]	U = 240.5	0.260

Continuous variables are presented as mean ± standard deviation (SD) or as median (Me) with interquartile range [IQR; Q1–Q3], based on their distribution. Abbreviations: 6MWT, 6-min walk test; cQTc, corrected QT interval to account for bundle branch block or biventricular pacing; EMA, erythrocyte membrane β-adrenoreactivity; IVS, interventricular septum; LVEF, left ventricle ejection fraction; LVESD, left ventricular end-systolic dimension; LVESV, left ventricular end-systolic volume; LVGLS, left ventricular global longitudinal strain; LVSI, left ventricular sphericity index; NTproBNP, N-terminal pro-B-type natriuretic peptide; pts, patients; t, Student’s t-statistic; TC, total cholesterol; U, inversion number criterion; VTA, ventricular tachyarrhythmia. *Between-group comparisons were performed using the t-test or Mann–Whitney U test for continuous variables, as appropriate.

**Table 3:** Selected baseline-to-12-month changes after CRT-D implantation.

Measure	Pts with VTA: Baseline → 12 m.	TS; *p* Within	Pts Without VTA: Baseline → 12 m.	TS; *p* Within	TS; *p* Baseline	TS; *p* 12 m.
HFFC III, n (%)	21 (55.2) → 0 (0.0)	MNT; <0.001	8 (25.0) → 0 (0.0)	MNT; 0.008	FET; 0.015	FET; 0.473
EuroQoL EQ-5D	61.5 [55.0; 67.0] → 80.0 [75.0; 85.0]	T = 37.5; <0.001	57.5 [54.0; 68.5] → 80.0 [70.0; 85.0]	T = 0.0; <0.001	U = 560.0; 0.575	U = 598.5; 0.915
MLWHFQ	56.0 [47.0; 69.0] → 16.5 [11.0; 30.0]	T = 13.0; <0.001	54.5 [41.0; 67.0] → 16.0 [9.5; 38.0]	T = 0.0; <0.001	U = 542.5; 0.443	U = 592.0; 0.855
QRS, ms	180.7 ± 19.8 → 144.0 ± 17.3	t = 11.92; <0.001	172.4 ± 21.1 → 139.7 ± 15.2	t = 8.87; <0.001	t = −1.677; 0.098	t = −1.089; 0.280
cQTc, ms	428.9 ± 26.7 → 425.9 ± 23.4	t = 0.61; 0.545	428.4 ± 26.9 → 429.0 ± 27.1	t = −0.09; 0.925	t = −0.066; 0.947	t = 0.500; 0.618
6MWT, m	280.0 [250.0; 350.0] → 450.0 [410.0; 470.0]	T = 0.0; <0.001	321.0 [299.5; 400.0] → 500.0 [445.0; 507.5]	T = 0.0; <0.001	U = 426.5; 0.033	U = 375.5; 0.006
EMA, %	31.4 [22.8; 48.3] → 29.2 [18.8; 42.7]	T = 394.0; 0.267	22.0 [15.8; 33.4] → 24.1 [17.9; 32.2]	T = 241.0; 0.667	U = 395.5; 0.012	U = 512.0; 0.260
NTproBNP, pg/mL	872.0 [257.0; 2598.0] → 872.0 [222.1; 2668.0]	T = 16.0; 0.779	419.6 [141.9; 1607.0] → 141.9 [109.0; 466.4]	T = 3.0; 0.062	U = 160.0; *p* = 0.338	U = 121.0; *p* = 0.043
IVS, mm	9.6 ± 1.4 → 10.0 ± 1.8	t = −2.53; 0.015	10.3 ± 1.4 → 10.7 ± 1.5	t = −3.06; 0.004	t = 2.274; 0.026	t = 1.895; 0.062
LVSI	0.68 ± 0.07 → 0.63 ± 0.09	t = 3.18; 0.003	0.65 ± 0.06 → 0.59 ± 0.07	t = 4.66; <0.001	t = −2.225; 0.029	t = −1.911; 0.060
LVESD, mm	60.2 ± 7.1 → 52.1 ± 11.6	t = 5.06; <0.001	56.7 ± 4.7 → 47.0 ± 10.2	t = 6.26; <0.001	t = −2.310; 0.024	t = −1.915; 0.059
LVESV, ml	175.0 [145.0; 210.0] → 116.0 [85.0; 167.0]	T = 38.0; <0.001	159.5 [129.5; 198.0] → 98.0 [67.5; 121.5]	T = 25.0; <0.001	U = 501.0; 0.209	U = 438.0; 0.045
LVEF, %	29.0 [25.0; 32.0] → 38.0 [33.0; 44.0]	T = 5.5; <0.001	30.0 [26.0; 35.0] → 42.5 [36.0; 52.5]	T = 5.5; <0.001	U = 506.5; 0.233	U = 465.5; 0.094
LVGLS, %	−7.8 [−10.1; −6.7] → −12.2 [−14.6; −9.9]	T = 3.0; <0.001	−9.0 [−11.2; −6.9] → −12.0 [−15.6; −8.6]	T = 17.0; <0.001	U = 240.5; 0.260	U = 350.0; 0.916

Continuous variables are presented as mean ± standard deviation (SD) or as median (Me) with interquartile range [IQR; Q1–Q3], based on their distribution. Categorical variables are reported as absolute frequencies (n) with relative frequencies (%). Abbreviations: 6MWT, 6-min walk test; cQTc, corrected QT interval to account for bundle branch block or biventricular pacing; CST, Chi-squared test; EMA, erythrocyte membrane β-adrenoreactivity; EuroQoL EQ-5D, European quality-of-life group questionnaire; FET, Fisher’s exact test; IVS, interventricular septum; LVEF, left ventricle ejection fraction; LVESD, left ventricular end-systolic dimension; LVESV, left ventricular end-systolic volume; LVGLS, left ventricular global longitudinal strain; LVSI, left ventricular sphericity index; MLWHFQ, Minnesota living with heart failure questionnaire; MNT, McNemar’s test; NTproBNP, N-terminal pro-B-type natriuretic peptide; pts, patients; t, Student’s t-statistic; T, Wilcoxon test statistic (sum of the lesser ranks); TC, total cholesterol; TS, test statistics; U, inversion number criterion; VTA, ventricular tachyarrhythmia. Between-group comparisons were performed using the t-test or Mann–Whitney U test for continuous variables, as appropriate, and Fisher’s exact test for categorical variables. Within-group comparisons were performed using the paired samples t-test or Wilcoxon signed-rank test statistic (calculated as the sum of the lesser ranks) for continuous variables, as appropriate, and McNemar’s test for categorical variables.

**Table 4:** Key logistic-regression and model-performance results.

Analysis	Association Estimate	Discrimination	Interpretation
EMA, univariable	OR 1.0426 per + 1%; 95% CI 1.0088–1.0774; *p* = 0.013	AUC 0.675 (0.552–0.782)	Modest discrimination
NYHA III, univariable	OR 3.71; 95% CI 1.33–10.32; *p* = 0.012	AUC 0.651	Candidate association
LVESD, univariable	OR = 1.10 per + 1 mm; *p* = 0.030	AUC 0.639 (0.515–0.750)	Structural marker
6MWT, univariable	OR 0.93 per + 10 m; *p* = 0.037	AUC 0.649	HL *p* = 0.048; calibration concern
EMA + LVESD, multivariable	EMA OR 1.0446 (*p* = 0.013); LVESD OR 1.1023 (*p* = 0.029)	AUC 0.748 (0.629–0.844)	Sensitivity 73.68%; specificity 71.87% at *p* = 0.5
Bootstrap-corrected model	EMA BCa CI 1.007–1.083; LVESD BCa CI 1.004–1.218	Corrected AUC 0.727 (0.612–0.835)	2000 resamples; corrected Brier 0.208

## Data Availability

The data presented in this study are available on request from the corresponding author. The data are not publicly available due to privacy or ethical restrictions.

## Abbreviations

The following abbreviations are used in this manuscript:

6MWT	6 min walk test
ADRB1	β1-adrenergic receptor
AUC	Area under the receiver-operating-characteristic curve
BCa	Bias-corrected and accelerated
CaMKII	Ca^2+^/calmodulin-dependent protein kinase II
cAMP	Cyclic adenosine monophosphate
CI	Confidence interval
CRT	Cardiac resynchronization therapy
CRT-D	Cardiac resynchronization therapy with defibrillation capability
ECG	Electrocardiography
EMA	Erythrocyte membrane β-adrenoreactivity
EuroQoL EQ-5D	European quality-of-life group questionnaire
GRK2	G protein-coupled receptor kinase 2
IVS	Interventricular septum
HF	Heart failure
HFrEF	Heart failure with reduced ejection fraction
HR	Hazard ratio
LV	Left ventricular
LVEF	Left ventricular ejection fraction
LVESD	Left ventricular end-systolic dimension
LVSI	Left ventricular sphericity index
MLWHFQ	Minnesota living with heart failure questionnaire
NT-proBNP	N-terminal pro-B-type natriuretic peptide
NYHA	New York Heart Association
OR	Odds ratio
PKA	Protein kinase A
ROC	Receiver-operating-characteristic
ROS	Reactive oxygen species
RyR2	Ryanodine receptor type 2
SERCA2a	Sarcoplasmic/endoplasmic reticulum calcium ATPase 2a
VIF	Variance inflation factor
VF	Ventricular fibrillation
VT	Ventricular tachycardia
VTA	Ventricular tachyarrhythmia

## References

[1] Muslimova E.F., Rebrova T.Y., Korepanov V.A., Akhmedov S.D., Afanasiev S.A. (2024). Betaadrenoreactivity of erythrocyte membranes and expression of beta1-adrenergic receptors of cardiomyocytes in patients with heart failure with different left ventricular ejection fraction. Sib. J. Clin. Exp. Med..

[2] Johnson D.M., Antoons G. (2018). Arrhythmogenic Mechanisms in Heart Failure: Linking beta-Adrenergic Stimulation, Stretch, and Calcium. Front. Physiol..

[3] Visca A., Sherazi S., Goldenberg I., McNitt S., Rao N., Rao N., Shah A., Aktas M., Kutyifa V., Zareba W. (2025). Predicting Ventricular Tachyarrhythmias in Patients With Left Ventricular Ejection Fraction Improvement Following Cardiac Resynchronization Therapy. Ann. Noninvasive Electrocardiol..

[4] Savarese G., Becher P.M., Lund L.H., Seferovic P., Rosano G.M.C., Coats A.J.S. (2023). Global burden of heart failure: A comprehensive and updated review of epidemiology. Cardiovasc. Res..

[5] Ran J., Zhou P., Wang J., Zhao X., Huang Y., Zhou Q., Zhai M., Zhang Y. (2025). Global, regional, and national burden of heart failure and its underlying causes, 1990–2021: Results from the global burden of disease study 2021. Biomark. Res..

[6] Goldenberg I., Aktas M.K., Zareba W., Tsu-Chau Huang D., Rosero S.Z., Younis A., McNitt S., Stockburger M., Steinberg J.S., Buttar R.S. (2024). QRS Morphology and the Risk of Ventricular Tachyarrhythmia in Cardiac Resynchronization Therapy Recipients. JACC Clin. Electrophysiol..

[7] Muslimova E.F., Rebrova T.Y., Sonduev E.L., Popova V.O., Kozlov B.N., Afanasyev S.A. (2024). Association of betaadrenoreactivity of erythrocyte membranes with the severity of chronic heart failure in patients with coronary heart disease. Complex Issues Cardiovasc. Dis..

[8] Goto T., Inden Y., Yanagisawa S., Tsurumi N., Miyamae K., Miyazawa H., Kondo S., Tachi M., Iwawaki T., Yamauchi R. (2025). A Practical Scoring System for Estimating Ventricular Arrhythmia Events in Patients with Cardiac Resynchronization Therapy for Primary Prevention. Int. Heart J..

[9] Higuchi K., Manne M., Tchou P., Baranowski B., Bhargava M., Callahan T., Chung M., Dresing T., Hussein A., Kanj M. (2025). Left ventricular mass as a modulator of ventricular arrhythmia risk and sex differences after CRT for nonischemic cardiomyopathy and LBBB. Heart Rhythm.

[10] Muslimova E.F., Korepanov V.A., Rebrova T.Y., Andreev S.L., Afanasiev S.A. (2024). Correlation of β-adrenergic reactivity of erythrocyte membranes with clinical characteristics of patients with coronary artery disease and HF with preserved and reduced ejection fraction. Russ. J. Cardiol..

[11] Benitah J.P., Pereira L., Perrier R., Mercadier J.J., Sabourin J., Gómez A.M. (2026). Local calcium dynamics and signalling in cardiomyocytes. Nat. Rev. Cardiol..

[12] Nibley P.C., Shenoy S.K. (2024). β-adrenergic receptor signaling mediated by β-arrestins and its potential role in heart failure. Curr. Opin. Physiol..

[13] Kaplan A., El-Samadi L., Zahreddine R., Amin G., Booz G.W., Zouein F.A. (2025). Canonical or non-canonical, all aspects of G protein-coupled receptor kinase 2 in heart failure. Acta Physiol..

[14] Kraft A.E., Bork N.I., Subramanian H., Pavlaki N., Failla A.V., Zobiak B., Conti M., Nikolaev V.O. (2024). Phosphodiesterases 4B and 4D Differentially Regulate cAMP Signaling in Calcium Handling Microdomains of Mouse Hearts. Cells.

[15] Tzimas C., Terrovitis J., Lehnart S.E., Kranias E.G., Sanoudou D. (2015). Calcium/calmodulin-dependent protein kinase II (CaMKII) inhibition ameliorates arrhythmias elicited by junctin ablation under stress conditions. Heart Rhythm.

[16] Mattiazzi A., Bers D.M. (2026). CaMKII in the Heart: From Homeostasis to Pathology. Annu. Rev. Physiol..

[17] Gao K., Wang W., Ling Y., Li B., Xing C., Li N., Yin X., Tao L., Li X., Qiu J. (2026). Structural and Functional Regulation of RyR2 in Cardiac Calcium Handling and Arrhythmogenesis. Biomedicines.

[18] Muslimova E., Rebrova T., Kondratieva D., Korepanov V., Sonduev E., Kozlov B., Afanasiev S. (2022). Expression of the β1-adrenergic receptor (ADRB1) gene in the myocardium and β-adrenergic reactivity of the body in patients with a history of myocardium infarction. Gene.

[19] Triposkiadis F., Briasoulis A., Kitai T., Magouliotis D., Athanasiou T., Skoularigis J., Xanthopoulos A. (2024). The sympathetic nervous system in heart failure revisited. Heart Fail. Rev..

[20] van Weperen V.Y.H., Ripplinger C.M., Vaseghi M. (2023). Autonomic control of ventricular function in health and disease: Current state of the art. Clin. Auton. Res..

[21] Arshad M.S., Kittipibul V., Fudim M. (2025). Sympathetic Nervous System in Heart Failure: Targets for Treatments. Curr. Hypertens. Rep..

[22] Parichatikanond W., Duangrat R., Kurose H., Mangmool S. (2024). Regulation of β-Adrenergic Receptors in the Heart: A Review on Emerging Therapeutic Strategies for Heart Failure. Cells.

[23] Yang B., Wang S.-Q., Yang H.-Q. (2024). β-adrenergic regulation of Ca2+ signaling in heart cells. Biophys. Rep..

[24] Mo W., Beard N.A., Gillette-Browne K.T., Hay K., Cheesman E., Dashwood A., Spratt M., Semmler A.B., Wong Y.W., Haqqani H. (2026). β1- and β2-adrenergic receptor mediated spontaneous contractions and ryanodine receptor channel opening in human failing heart. Naunyn-Schmiedeberg’s Arch. Pharmacol..

[25] Atabekov T.A., Batalov R.E., Rebrova T.Y., Krivolapov S.N., Muslimova E.F., Khlynin M.S., Afanasiev S.A., Popov S.V. (2022). Ventricular tachycardia incidence and erythrocyte membranes β-adrenoreactivity in patients with implanted cardioverter-defibrillator. Pacing Clin. Electrophysiol..

[26] Sager G. (1982). Receptor binding sites for β-adrenergic ligands on human erythrocytes. Biochem. Pharmacol..

[27] Bree F., Gault I., d’Athis P., Tillement J.P. (1984). Beta adrenoceptors of human red blood cells, determination of their subtypes. Biochem. Pharmacol..

[28] Falcão-Pires I., Ferreira A.F., Trindade F., Bertrand L., Ciccarelli M., Visco V., Dawson D., Hamdani N., Van Laake L.W., Lezoualc’H F. (2024). Mechanisms of myocardial reverse remodeling and its clinical significance: A scientific statement of the ESC Working Group on Myocardial Function. Eur. J. Heart Fail..

[29] Chung M.K., Patton K.K., Lau C.-P., Forno A.R.D., Al-Khatib S.M., Arora V., Birgersdotter-Green U.M., Cha Y.-M., Chung E.H., Cronin E.M. (2023). 2023 HRS/APHRS/LAHRS guideline on cardiac physiologic pacing for the avoidance and mitigation of heart failure. Heart Rhythm.

[30] Pilecky D., Duray G.Z., Elsner D., Israel C.W., Erath-Honold J.W., Vamos M. (2022). Association between electrical and mechanical remodeling after cardiac resynchronization therapy: Systematic review and meta-analysis of observational studies. Heart Fail. Rev..

[31] Allison J.D., Biton Y., Mela T. (2022). Determinants of Response to Cardiac Resynchronization Therapy. J. Innov. Card. Rhythm Manag..

[32] Theofilis P., Iliakis P., Karanikola A.-E., Botis M., Mavromoustakou K., Xydis P., Ktenopoulos N., Karakasis P., Leontsinis I., Chrysohoou C. (2026). Pleiotropic Effects of Cardiac Resynchronization Therapy on Cardiometabolic Modulation in Heart Failure. Medicina.

[33] Elliott M.K., Strocchi M., Mehta V.S., Wijesuriya N., Mannakkara N.N., Jackson T., Pereira H., Behar J.M., Bishop M.J., Niederer S. (2022). Dispersion of repolarization increases with cardiac resynchronization therapy and is associated with left ventricular reverse remodeling. J. Electrocardiol..

[34] Ueda N., Ishibashi K., Noda T., Oka S., Miyazaki Y., Shimamoto K., Wakamiya A., Nakajima K., Kamakura T., Wada M. (2024). Implications of ventricular arrhythmia after cardiac resynchronization therapy. Heart Rhythm.

[35] Roshanbinfar K., Schiffer M., Carls E., Angeloni M., Koleśnik-Gray M., Schruefer S., Schubert D.W., Ferrazzi F., Krstić V., Fleischmann B.K. (2024). Electrically Conductive Collagen-PEDOT:PSS Hydrogel Prevents Post-Infarct Cardiac Arrhythmia and Supports hiPSC-Cardiomyocyte Function. Adv. Mater..

[36] Zeppenfeld K., Tfelt-Hansen J., de Riva M., Winkel B.G., Behr E.R., Blom A.N., Charron P., Corrado D., Dagres N., de Chillou C. (2022). 2022 ESC Guidelines for the management of patients with ventricular arrhythmias and the prevention of sudden cardiac death. Eur. Heart J..

